# Mid-term clinical and radiological outcomes of arthroscopic repair of isolated and combined subscapularis tears: A single-center experience

**DOI:** 10.5152/j.aott.2021.20420

**Published:** 2021-11-01

**Authors:** Recep Kurnaz, Selim Ergün, Murat Aşçı, Umut Akgün, Taner Güneş

**Affiliations:** 1 Clinic of Orthopedics and Traumatology, Acıbadem Eskişehir Hospital, Eskişehir, Turkey; 2 Clinic of Orthopedics and Traumatology, Kartal Dr. Lütfi Kırdar City Hospital, İstanbul, Turkey; 3 Department of Orthopedics and Traumatology, Acıbadem Mehmet Ali Aydınlar University, School of Medicine, İstanbul, Turkey

**Keywords:** Subscapularis, Double-row repair, Single-row repair, Rotator cuff, Arthroscopy

## Abstract

**Objective:**

The aim of this study was to evaluate the mid-term clinical and radiological results of patients who underwent arthroscopic subscapularis (SSC) tendon repair and to address the possible effect of repair technique(single or double row), tear pattern, and concomitant procedures among supraspinatus tears and long head of biceps tendon (LHBT) pathologies on outcomes and failure parameters.

**Methods:**

45 patients (24 female; mean age = 55.9 years, age range = 37 - 78) who underwent arthroscopic repair of an SSC tear between January 2009 and December 2016 were retrospectively identified and included in the study. Pre- and postoperative internal rotation strength and shoulder joint range of motion angles were measured. Clinical outcomes were assessed by Visual Analog Scale (VAS), American Shoulder and Elbow Surgeons (ASES), Constant-Murley, Rotator cuff-quality of life (RC-QoL), and University of California Los Angles (UCLA) scores preoperatively and at the final follow-up.

**Results:**

The mean follow-up was 45.2 (range = 36-104) months. 14 patients had isolated SSC tears. The mean preoperative VAS, ASES, Constant-Murley, RC-QoL, and UCLA scores for all patients were respectively 8.6, 21.2, 24, 28.9, and 12. Significant improvement was observed in each clinical outcome at the final follow-up: 0.96, 88.4, 86.4, 90.2, and 32.2, respectively. Improvement in outcome scores was more prominent in patients with Lafosse grade I and II SSC tendon tears repaired by single-row technique and in patients with concomitant supraspinatus tendon repairs. The mean preoperative internal rotation strength according to the Oxford scale was 3.4 (± 0.6) / 5 and raised to 4.7 (± 0.4) / 5 at the final follow-up (*P* <.001). Although concomitant biceps interventions significantly improved the outcome scores; this improvement was not clinically significant. Failure was only seen in 6 patients with high-grade (Lafosse III or IV) tears.

**Conclusion:**

Significant improvement in clinical outcomes and lower failure ratios were more prominent in patients with Lafosse grade I or II tears than grade III or IV. Concomitant biceps interventions made a positive contribution to the clinical outcome. Early diagnosis and repair seem to be advantageous before low-grade SSC tendon tears turn into high-grade tears.

**Level of Evidence:**

Level IV, Therapeutic Study

## Introduction

The subscapularis (SSC) muscle plays an essential role in internal rotation and dynamic anterior stability of the glenohumeral joint.^1^ Tears of SSC tendon can be seen after an acute hyperextension or external rotation and abduction injury in young patients. In the elderly, tendon degeneration and anterior dislocation are the most common etiologic factors.^2^

Surgical repair of the SSC tendon is essential to regain the internal rotation strength and dynamic anterior stability. However, it is not a common procedure among shoulder arthroscopy procedures. When compared with superior rotator cuff lesions, isolated SSC tendon tears are only 4-5% of all rotator cuff tears.^3,4^ In autopsy and cadaveric studies, SSC tear rate has been shown to be between 3% and 13%.^5^ Unlikely, they are considerably more common when present with a concomitant posterosuperior cuff (supraspinatus and/or infraspinatus) tear; 27-35% of all rotator cuff tears.^6-^^8^

SSC tears are also called “hidden tears” because they can be overlooked in preoperative radiological evaluation, even during arthroscopy. After Burkhart reported first arthroscopic SSC repair technique in 2002, arthroscopic repair became more popular than open repair with the advantage of detecting those “hidden tears” of the SSC tendon.^9^

Studies that report the clinical and radiological outcome after arthroscopic SSC repairs are increasing in number.^2,4,6–23^ Although studies mostly report isolated SSC tears, combined other rotator cuff and biceps pathologies frequently co-exist. Besides, tear grades and repair techniques, such as single- or double-row repair, differ between those studies.

The purpose of this study was to (1) analyze the postoperative clinical outcome scores, internal rotation strength and retear rates among arthroscopically repaired SSC tendon tears, and (2) compare the effect of arthroscopic technique (single or double row), SSC tear pattern and concomitant procedures among supraspinatus tears and Long Head of Biceps Tendon (LHBT) pathologies over outcome and failure parameters. We hypothesized that arthroscopic repair by single- or double-row technique is a reasonable option to treat SSC tendon tears, either isolated or with concomitant supraspinatus tendon tears. Moreover, concomitant biceps tendon interventions will provide a better outcome.

## Materials and Methods

Patients who underwent arthroscopic repair of a SSC tear between January 2009 and December 2016 were included in this study. Inclusion criteria consisted of (I) having a SSC tendon tear with or without concomitant supraspinatus tendon tears, (II) concomitant LHBT pathologies, and (III) at least a 2-year follow-up period. Patients who had infraspinatus and/or teres minor tendon tears, irreparable rotator cuff tendon tears (any massive retracted SS or SSC tendon tear with Goutallier Grade 3 or higher muscle fatty degeneration) necessitating tendon transfer, frozen shoulder findings, cervical vertebra-related complaints, degenerative arthritic findings in the glenohumeral joint and history of previous fracture or surgery on the same shoulder were excluded. This study was approved by the local institutional ethics committee (approval number: 2020-02/03), and all patients signed informed consent forms regarding operative treatment and follow-up investigations.

### Clinical and radiological evaluation

ll clinical examinations were performed by the senior author. Active range of motion was measured by goniometric assessment for forward elevation (flexion), external and internal rotation. Clinical outcome was assessed by Visual Analog Scale (VAS), American Shoulder and Elbow Surgeons (ASES), Constant–Murley, Rotator Cuff-Quality of Life (RC-QoL) and University of California Los Angles (UCLA) scores. All these clinical outcome tests were asked to patients preoperatively, and at the last follow-up. Results were compared not only for statistical significance but also for the clinical significance as stated in the literature for each scoring system.^24–26^

**Figure 1.**
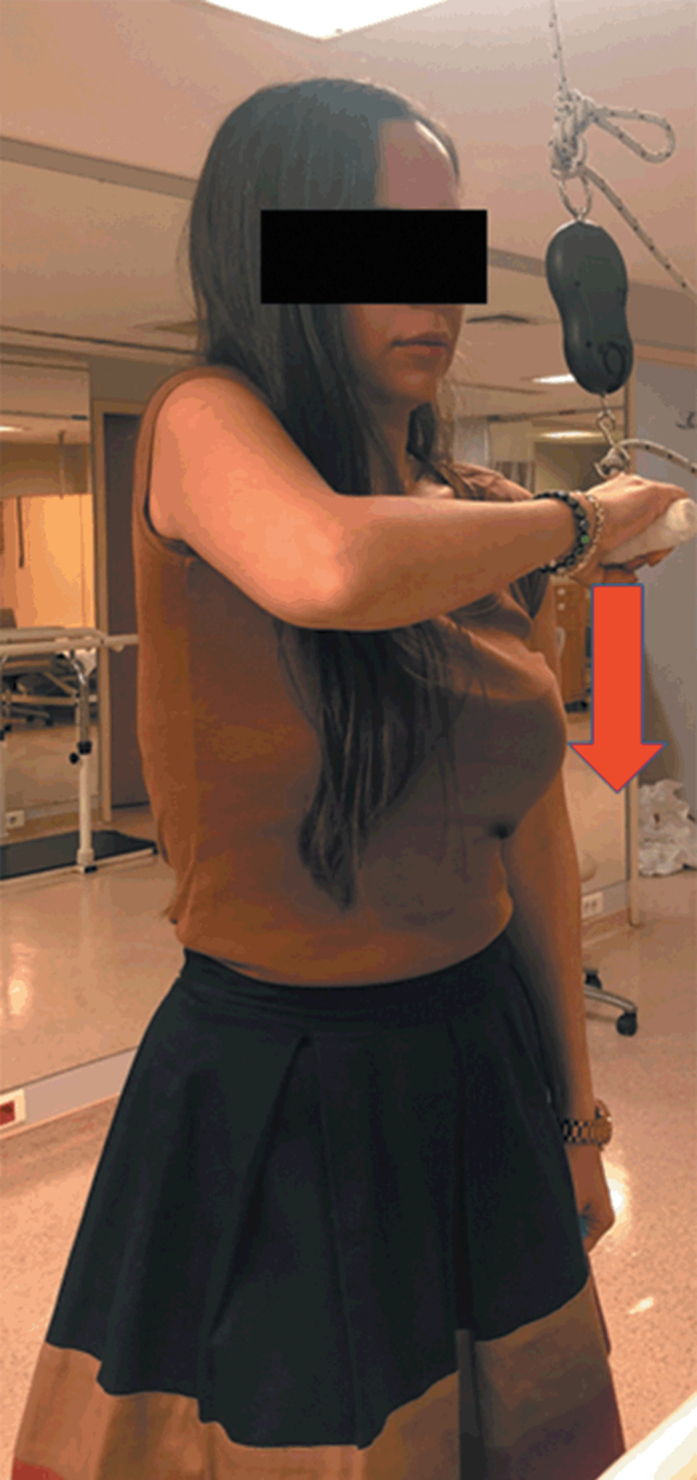
Strength measurement by a digital gauge (Lutron FG-5005, Taiwan) while patient performing the bear hug test.

Pre- and postoperative strength of the SSC muscle was measured by Oxford Scale (Medical Research Council) which grades muscle strength on a 0 to 5 scale. Postoperative strength was also measured by a digital gauge (Lutron FG-5005, Taiwan) while patient performing the bear hug test at postoperative the 6^th^ month (Figure 1)^17^.

**Figure 2.**
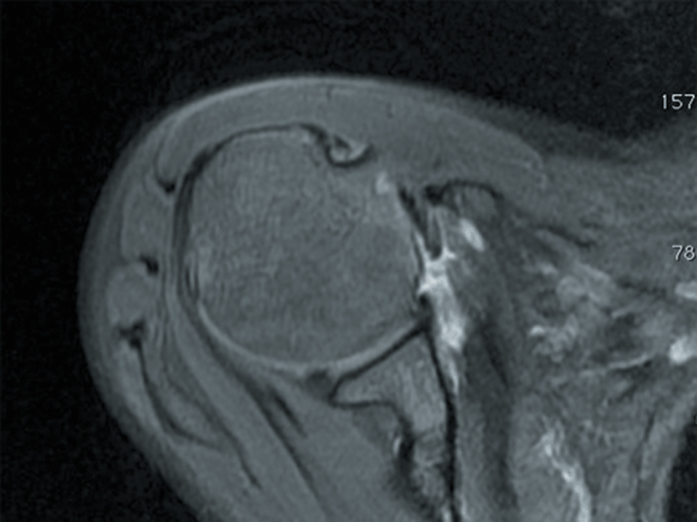
Axial MRI view of a patient with SSC tendon tear.

Preoperative MRI was routinely seen to diagnose and classify the grade of SSC tendon tear (Figure 2) and check the additional pathologies regarding rotator cuff and biceps tendons. While there was no sign of SSC tear in the MR imaging of some patients, preoperatively it was found that SS tendon tear was accompanied by a SSC tendon tear, and tear grading was made according to arthroscopic findings. Radiological control of the repair integrity by MRI was not performed routinely, only patients with the suspect of retear were radiologically re-examined.

### Operative technique

All patients were operated under general anesthesia, in the lateral decubitus position and by the same surgeon. Standard posterior, anteroinferior, anterosuperior and lateral portals were used. Diagnostic arthroscopy was performed via these portals. LHBT was arthroscopically evaluated for any injury, and the tendon was graded according to the Lafosse classification system; Grade 0: normal tendon, Grade 1: partial (less than 50% of tendon erosion or loss of the tendon) and Grade 2: >50% erosion or loss of the tendon.^27^

**Figure 3.**
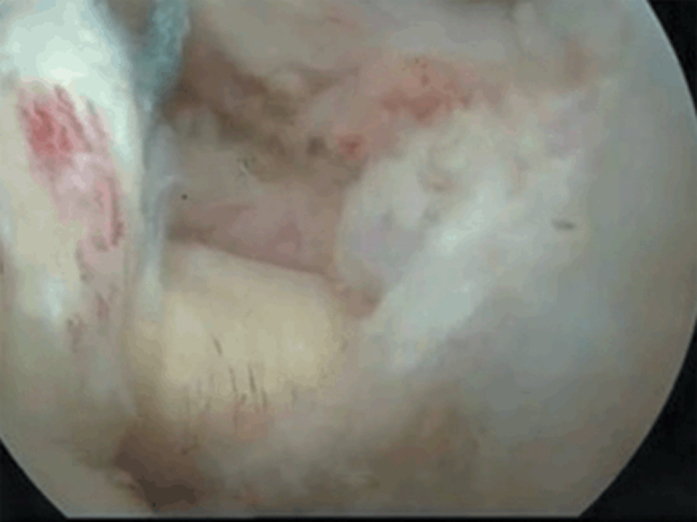
Arthroscopic image from posterior portal demonstrating secure fixation of the tendon to lesser tuberosity footprint by single-row repair technique.

**Figure 4. a, b.**
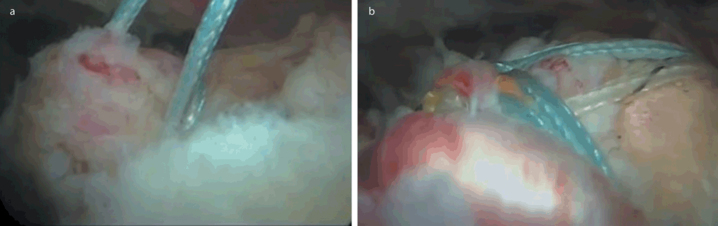
Arthroscopic image from posterior portal demonstrating (a) temporary traction suture passed through the SSC tendon. (b) Arthroscopic image from anterior portal demonstrating secure fixation of the tendon to lesser tuberosity footprint by double-row repair technique.

After circumferential release of the SSC tendon, footprint was debrided by shaver at the lesser tuberosity. Single- or double-row repair was chosen according to the size of the tear.^4,28^ Single-row repair was chosen for patients with Lafosse grade I or II SSC tears and performed by 5.5 mm titanium screw anchors (Corkscrew FT II, Arthrex, USA) inserted from the anterosuperior portal, from inferior to superior (Figure 3). For larger tears, a temporary traction suture was passed through the tendon, and then, double-row repair was performed by 4.75 mm PEEK anchor (SwiveLock C, Arthrex, USA) for the medial row and 5.5 mm PEEK knotless anchors (SwiveLock SP^®^, Arthrex, USA) for the lateral row^4,28^ (Figure 4). In cases of concomitant supraspinatus tears, repair by double-row technique was performed with the same anchors over its footprint at the greater tuberosity. SSC tendon was primarily repaired in patients with concomitant supraspinatus tendon tears.

### Postoperative rehabilitation

All patients were immobilized with an arm sling (in internal rotation, without an abduction pillow) during the postoperative 6-week period. In the early postoperative period, pendulum exercises, passive internal rotation and abduction movements were started under the supervision of a physiotherapist. Passive ER to neutral and pain-free forward flexion were permitted during the first 6 weeks after surgery. After 6 weeks, unrestricted active-assisted forward flexion, abduction and rotations were allowed. Strengthening of the rotator cuff was started at week 12.

### Statistical analysis

Descriptive analyzes were conducted to inform about the general characteristics of the study groups. Data of continuous variables were as mean ± standard deviation. Data on categorical variables are given in *n* (%). Paired *t* and Chi-Square used to evaluate the data collected. *P* values were considered to be statistically significant when they were calculated less than 0.05. The Mann–Whitney *U*-test was performed to compare differences in clinical outcome scores according to presence of concomitant supraspinatus tendon tears, biceps pathologies and according to repair technique (single or double row). Ready-to-use statistical software was used in the calculations (IBM SPSS Statistics 19, SPSS Inc., an IBM Co., Somers, NY, USA).

## Results

Sixty-two patients were arthroscopically operated with the diagnosis of SSC tendon tear. Among these patients, 11 were excluded due to concomitant infraspinatus or teres minor tendon involvement, and 6 patients refused participation. A total of 45 patients were included in the study. Twenty-four patients were female and the mean age was 55.9 ± 9 years (range 37-78). Thirty-eight patients had the tear in his/her dominant arm (84%) (Table 1). In most patients, SSC tear was diagnosed radiologically on preoperative MRI (37 patients), but preoperative diagnosis was done in 8 patients whose preoperative magnetic resonance images were inconsistent with a SSC tear. All patients had a decreased strength in the internal rotation of the shoulder.

Most patients (*n* = 30) had a history of trauma. Isolated SSC tears were detected in 14 patients, remaining 31 had concomitant supraspinatus tendon tears. The mean time interval between the onset of the symptoms and the operation was 14.5 ± 11 (range 2-48) months (Table 1).

Single-row repair technique was chosen for 14 patients who had a Lafosse grade I or II SSC tear, and the remaining patients had grade III or IV tear patterns and were repaired by double-row repair technique (Table 2). The mean follow-up period was 45.2 ± 16 (36-104) months (Table 1); 46.66 ± 19.9 (32-104) for patients with grades I and II SSC tears and 44.56 ± 9.08 (30-86) for patients with grades III and IV SSC tears (*P* > 0.05).

Mean preoperative VAS, ASES, Constant–Murley, RC-QoL and UCLA scores for all patients were 8.6, 21.2, 24, 28.9 and 12, respectively. At the last follow-up, same scores were found to be as 0.96, 88.4, 86.4, 90.2 and 32.2, respectively (Table 3). Improvements in all clinical scores were clinically significant. In cases with concomitant supraspinatus cuff repair, all scores except UCLA were found to be significantly better than the cases with isolated SSC tear (Table 4). However, this improvement was clinically significant for only Constant–Murley scores.

The LHBT was found to be normal in 30 cases (66.6%) and pathologic (inflammatory changes, Lafosse grades 1 and 2 injuries) in 15 (33.3%). LHBT pathologies were more common (13 of 15 patients) among patients with accompanying supraspinatus tendon tear. Only 3 patients were treated by arthroscopic suprapectoral tenodesis procedure, whereas tenotomy was performed in 12 cases. LHBT undergone tenotomy or tenodesis significantly improved RC-QoL, Constant–Murley, and VAS scores better than the patients with normal LHBT; however, this difference was not clinically significant (Table 5).

Fourteen patients with Lafosse grade I or II tears that were repaired by single-row technique showed significantly better postoperative VAS, Constant–Murley and RC-QoL scores than grades III and IV tears treated by double-row technique (Table 6). However, this improvement was clinically significant for only Constant–Murley scores. Groups were found to be randomized regarding concomitant supraspinatus tear and biceps pathologies (Table 6).

The mean preoperative internal rotation strength according to Oxford Scale was 3.4 (±0.6)/5 and raised to 4.7 (±0.4)/5 at the final follow-up (*P* < 0.001). Bear hug test was performed postoperatively and quantitatively measured by a digital dynamometer. Mean value was found to be 49.4N in the operated shoulder and 59.8N for the intact shoulder (82% strength of the intact shoulder).

The mean preoperative flexion and external rotation angles were 138.2° (±24.5) and 28.8° (±7.1), which raised to 166.1° (±15.4) and 39.6° (±8.6) at the final follow-up (*P* < 0.001). Mean internal rotation ability level raised from the preoperative level of L4 to the postoperative level of L2.

Six patients (13%) had decreased strength and ongoing pain. Postoperative MRI showed that they had a reteared SSC tendon (five had Sugaya grade III, and one had grade IV retear). All these six patients had an isolated SSC tendon tear and were repaired by double-row technique, by the way they were Lafosse grade III or IV tears. They all refused reoperation and are followed up conservatively. Any other minor or major complication was not reported.

## Discussion

Mid-term follow-up results demonstrated that there was significant improvement in outcome scores of all patients after arthroscopic repair of SSC tendon tears, either isolated or with concomitant supraspinatus tendon repairs. Improvement was more prominent in patients with low-grade partial SSC tears repaired by single-row technique when compared to higher grade tears repaired by double-row technique. Failure of the SSC repair was only seen in patients those with high-grade tears. Concomitant biceps interventions were also found to improve postoperative outcome, confirming our hypothesis.

It is known that tears of the subscapularis tendon often necessitate addressing the LHBT surgically because of its anatomic neighbourhood with the soft tissue sling covering the bicipital groove. Edwards et al. noticed more favourable outcome results with concomitant tenotomy or tenodesis of the LHBT in their patients with open SSC tendon repairs; furthermore, they recommended LHBT tenotomy or tenodesis for all patients undergoing subscapularis tendon repair, independent of the preoperative condition of the biceps tendon.^29^

Open repair was regarded as the gold standard for the treatment of SSC tendon tears.^30^ However, recent studies on newer arthroscopic techniques have shown promising results.^2,12,15,16,20,21,23,31^ In 2002, Burkhart first reported the arthroscopic SSC tendon repair results, then Bennet in 2003.^8,9^ Both studies used single-row repair and reported improved clinical results; however, they had small sample size, few outcome parameters and lack objective strength measurements. However, concomitant biceps interventions had been performed but not taken into consideration for the effect on outcomes.

Recent studies begun to report the SSC repair results by considering the effect of concomitant interventions regarding LHBT and other rotator cuff pathologies, SSC tendon repair technique, SSC tendon tear pattern and muscle fatty degeneration. Among these studies, only Seppel et al. considered the effect of concomitant LHBT interventions on outcome scores and noticed that LHBT intervention did not correlate with inferior or superior functional outcome results.^2^ It was also reported that there was no correlation with the SSC tendon tear pattern and clinical outcome scores.

In a review of arthroscopic repair of the isolated SSC tendon tears, Saltzman et al. stated that there was insufficient data to investigate the correlation of outcome scores with the repair technique (single or double row) or concomitant LHBT intervention.^31^ Although patients treated with double-row technique had superior results and low retear rate, they noticed an apparent selection bias of double-row repairs being performed in more severe tear patterns. In the present study, we noticed the same selection bias because we repaired all grades III and IV tears by double-row technique. However, we found inferior outcome results and high retear rate among those patients and we think that this difference should be attributed to the tear pattern, rather than the double-row technique. Further data are necessary before a conclusion can be drawn.

In the literature, few studies have evaluated the results of SSC tear repair as a part of anterosuperior rotator cuff tear (with concomitant supraspinatus tear) and compared the outcome results with isolated SSC tendon repairs.^12,20,22^ According to the results of these studies, there were no significant differences among outcome scores, internal rotation strength and range of motion in between groups. Although concomitant biceps interventions^12,20,22^ and different SSC repair techniques^12^ were performed, these additional procedures were not randomized. In the present study, SSC tendon tears were isolated in 14 patients and with concomitant supraspinatus tendon tear in 31 patients. We found that both groups were randomized according to repair technique but not for concomitant biceps interventions. Even though significantly increased outcome scores were found in patients with concomitant supraspinatus repairs, the majority of the biceps tenodesis and tenotomy procedures (13 vs. 2 patients) had been performed in those patients. At this stage, which the concomitant procedure, whether supraspinatus repair or biceps intervention, induced the increase in outcome results, is unclear. In our opinion, concomitant biceps intervention is very determinative at this point. As previously discussed, more favourable outcome results should be expected with concomitant tenotomy or tenodesis of the LHBT, but further research is necessary.

Among the studies that evaluated SSC tear repair as a part of anterosuperior rotator cuff tear, a recent study by Meshram et al. evaluated the risk factors for retear complication of SSC tendon repair.^20^ They found that the optimal cutoff values for the risk of retear was 19 mm retraction and 16 mm superoinferior tear dimension of the tendon tear, and grade III or IV fatty degeneration of SSC muscle. Similar to their findings, we found decreased outcome results in patients with Lafosse grades III and IV tears when compared to patients with grades I and II, and all reteared tendons were preoperatively grade III or IV as well.

Failure rates for SSC tendon repair were similar among studies. Lafosse et al. found the retear rate as 13% in patients with arthroscopic isolated SSC tendon repair.^28^ Retear complication was only reported in patients with preoperative grades III and IV tears that had been repaired by double-row technique.^28^ In the present study, we also found the retear rate as 13%, and we reported this complication only in patients with grades III and IV tears repaired by double-row technique. According to these findings, double-row repair might appear as a risk factor for retear; however, we think that the principle risk factor is the tear pattern. Findings of Warner et al. and Meshram et al. support this theory.^20,32^ According to their results, retear is most commonly seen in retracted grade IV tears, with fatty degenerated muscles.

This study has some limitations that must be considered. First, the study design was retrospective in nature. Second, there is no information about fatty degeneration of rotator cuff muscles, but preoperative delay between the onset of symptoms and the surgery and the incidence of a traumatic history were questioned and found to be randomized between the groups. Third, radiological confirmation of postoperative SSC tendon structural integrity was not routine for all patients, which might have contributed to the selection bias.

Nonetheless, this study has certain strengths. We have evaluated one of the largest cohort of patients who underwent arthroscopic repair for isolated SSC and concomitant supraspinatus tendon tears. Furthermore, we compared the clinical outcomes in both groups using five different pain and outcome scores, strength and ROM measurements. To the best of our knowledge, this is the first study that evaluated the effect of tear pattern, repair technique, concomitant supraspinatus tendon repair and biceps interventions on clinical outcome of patients with arthroscopic SSC tendon repair.

In conclusion, the current data suggest that significant improvement in clinical outcomes and lower failure ratios were more prominent in arthroscopic repair of low-grade SSC tears. Concomitant biceps interventions made a positive contribution to the clinical outcome. Early diagnosis and repair seems advantageous before low-grade SSC tendon tears turn into a high-grade tear.

**Highlights**

- Lafosse grade I or II subscapularis tendon tears respond to arthroscopic single-row repair better than grade III or IV tears repaired by double-row technique.
- Treatment of subscapularis tendon tears necessitates biceps tendon interventions.
- Early diagnosis and repair of the partial subscapularis tendon tears is crucial.

## Figures and Tables

**Table 1.** Descriptive Analysis of the 45 Patients

Mean age (years)	55.9 ± 9 (range 37-78)
Gender	24 female, 21 male
Dominant arm	38 patients
Etiology of the SSC tear	Traumatic in 30 patients, atraumatic in 15
SSC tendon tear grade	Grades I and II: 14 patients Grades III and IV: 31 patients
Concomitant supraspinatus tear repair	31 (68.8%) patients
Concomitant biceps pathology	15 patients
Mean time interval between onset of the symptoms and the operation (months)	Mean: 14.5 ± 11 (range 2-48) 7 patients ≤3 months 4 patients 3-6 months 21 patients 6-12 months 12 patients >12 months
Mean follow-up period (months) (all patients)	45.2 ± 16 (30-104)

**Table 2.** Classification of SSC Tendon Tear According to Lafosse Classification System^16^

Lafosse Grade	SSC Tear
I	Partial tear of superior 1/3
II	Complete tear of superior 1/3
III	Complete tear of superior 2/3
IV	Complete tear of the tendon, but head centered and fatty degeneration of the SSC muscle ≤stage 3
V	Complete tear, eccentric head, subcoracoid impingement and fatty degeneration of the SSC muscle ≥stage 3

**Table 3.** Pre- and Postoperative Clinical Outcome Scores of the 45 Patients

	Preoperative	Postoperative	*p*
VAS	8.6 ± 0.9	0.96 ± 0.8	*<0.001*
ASES	21.2 ± 4.9	88.4 ± 7.3	*<0.001*
Constant–Murley	24 ± 5.8	86.4 ± 6.1	*<0.001*
RC-QoL	28.9 ± 5.7	90.2 ± 2.7	*<0.001*
UCLA	12 ± 2.2	32.2 ± 1.8	*<0.001*

**Table 4.** Pre- and Postoperative Clinical Outcome Scores According to Concomitant Supraspinatus Tendon Repair

		Isolated SSC Tear and Repair (*n* = 14) (2 Biceps Pathology) (5 Single-Row and 9 Double-Row SSC Repair)	SSC + SS Tear and Repair (*n* = 31) (13 Biceps Pathology) (9 Single-Row and 22 Double-Row SSC Repair)	*P*
Age		56.56 ± 11.22	55.78 ± 8.59	0.82
Onset (months)		11.33 ± 2	15.39 ± 13.23	0.368
Follow up (months)		45 ± 16.62	45.33 ± 16.84	0.951
Trauma		9 patients	21 patients	0.704
Biceps pathology		2 patients	13 patients	0.068
Low-grade tear (single-row repaired)		5 patients	9 patients	0.654
VAS	Preoperative	8 ± 0	8.81 ± 0.95	**0.003**
	Postoperative	**1.89 ± 0.33**	**0.72 ± 0.7**	<**0.001**
ASES	Preoperative	23.33 ± 2.5	20.69 ± 5.23	0.08
	Postoperative	**80 ± 4.33**	**90.44 ± 6.39**	<**0.001**
Constant–Murley	Preoperative	22.67 ± 2	24.42 ± 6.42	0.326
	Postoperative	**81 ± 1.5**	**87.83 ± 6.11**	<**0.001**
RC-QoL	Preoperative	**32.99 ± 4.13**	**27.34 ± 5.67**	**0.002**
	Postoperative	**87.6 ± 1.24**	**90.84 ± 2.6**	<**0.001**
UCLA	Preoperative	11.67 ± 0.71	12.03 ± 2.48	0.599
	Postoperative	31.56 ± 2.3	32.42 ± 1.7	0.167

**Notes:** Bold values are only statistically significant; bold and underlined values are significant both statistically and clinically.

**Table 5.** Preoperative and Postoperative Clinical Outcome Scores According to Existing Biceps Tendon Pathology

		LHBT Intact (*n* = 30) (18 Concomitant SS Cuff Tear, 12 Isolated SSC Tear) (9 Single-Row and 21 Double-Row SSC Repair)	LHBT Tenotomy or Tenodesis (*n* = 15) (13 Concomitant SS Cuff Tear, 2 Isolated SSC Tear) (5 Single-Row and 10 Double-Row SSC Repair)	*P*
Age		54.5 ± 9.84	58.8 ± 6.56	0.134
Onset (months)		14.8 ± 10.37	14.13 ± 15.01	0.862
Follow up (months)		42.8 ± 11.47	50.2 ± 23.57	0.161
Trauma		19 patients	11 patients	0.502
Concomitant SS tear		18 patients	13 patients	0.068
Low-grade tear (single-row repaired)		9 patients	5 patients	0.819
VAS	Preoperative	8.77 ± 0.82	8.4 ± 1.06	*0.203*
	Postoperative	**1.13 ± 0.73**	**0.6 ± 0.83**	***0.034***
ASES	Preoperative	21.5 ± 4.18	20.67 ± 6.23	*0.599*
	Postoperative	88.67 ± 6.56	87.73 ± 8.91	*0.69*
Constant–Murley	Preoperative	**22.67 ± 4.11**	**26.87 ± 7.7**	***0.02***
	Postoperative	**84.52 ± 4.5**	**90.13 ± 7.29**	***0.002***
RC-QoL	Preoperative	28.36 ± 5.49	28.68 ± 6.62	*0.864*
	Postoperative	**89.44 ± 2.55**	**91.69 ± 2.47**	***0.007***
UCLA	Preoperative	11.57 ± 1.72	12.73 ± 2.94	*0.101*
	Postoperative	32.33 ± 1.79	32.07 ± 1.98	*0.66*

**Table 6.** Preoperative and Postoperative Clinical Outcome Scores According to Tear Grade and Repair Technique

		Lafosse Grades I and II Single-Row Repair (*n* = 14) (5 Biceps Pathology) (9 SS Cuff Repair)	Lafosse Grades III and IV Double-Row Repair (*n* = 31) (10 Biceps Pathology) (22 SS Cuff Repair)	*P*
Age		52.5 ± 8.19	56.69 ± 8.86	0.144
Onset (months)		14.86 ± 13.58	14.62 ± 11.75	0.952
Follow up (months)		44.5 ± 17.91	44.69 ± 14.78	0.97
Trauma		10 patients	20 patients	0.648
Biceps pathology		5 patients	10 patients	0.82
Concomitant SS tear		9 patients	22 patients	0.654
VAS	Preoperative	8.36 ± 1.01	8.83 ± 0.85	0.113
	Postoperative	**0.57 ± 0.65**	**1.07 ± 0.8**	**0.047**
ASES	Preoperative	19.29 ± 4.32	22.24 ± 5.1	0.067
	Postoperative	91.79 ± 3.72	87.62 ± 7.67	0.06
Constant–Murley	Preoperative	28.21 ± 7.53	22.34 ± 3.78	0.001
	Postoperative	**92.23 ± 4.44**	**84.07 ± 5.22**	**<0.001**
RC-QoL	Preoperative	29.22 ± 5.64	28.21 ± 6.16	0.604
	Postoperative	**92.87 ± 1.35**	**89.03 ± 2.34**	**<0.001**
UCLA	Preoperative	12.57 ± 2.77	11.72 ± 2	0.249
	Postoperative	32.57 ± 1.6	32.17 ± 1.91	0.499

Bold values are only statistically significant; bold and underlined values are significant both statistically and clinically.
